# 磺酸化磁性氮化碳固相萃取-超高液相色谱-串联质谱筛检淡水鱼中孔雀石绿和隐色孔雀石绿

**DOI:** 10.3724/SP.J.1123.2022.12009

**Published:** 2023-08-08

**Authors:** Erqiong MENG, Qixun NIAN, Feng LI, Qiuping ZHANG, Qian XU, Chunmin WANG

**Affiliations:** 1.东南大学公共卫生学院营养与食品卫生学系,江苏 南京 210009; 1. Department of Nutrition and Food Hygiene, School of Public Health, Southeast University, Nanjing 210009, China; 2.苏州市疾病预防控制中心, 江苏 苏州 215100; 2. Suzhou Center for Disease Control and Prevention, Suzhou 215100, China; 3.东南大学环境与医学工程教育部重点实验室,江苏 南京 210009; 3. Key Laboratory of Ministry of Environment and Medical Engineering, Southeast University, Nanjing 210009, China

**Keywords:** 磁性固相萃取, 磺酸化磁性氮化碳, 超高效液相色谱-串联质谱, 孔雀石绿, 隐色孔雀石绿, magnetic solid-phase extraction(MSPE), sulfonated magnetic graphite carbon nitride (S-MGCN), ultra performance liquid chromatography-tandem mass spectrometry (UPLC-MS/MS), malachite green (MG), leucomalachite green (LMG)

## Abstract

孔雀石绿(MG)及其代谢产物隐色孔雀石绿(LMG)在水产品中禁止检出,但违规使用行为屡禁不止,淡水鱼为抽检不合格率最高的水产品,因此,淡水鱼中MG和LMG的灵敏筛检对水产品食用安全非常重要。该工作研制了磺酸化磁性氮化碳(S-MGCN)材料,在考察其作为优良的磁性固相萃取(MSPE)吸附剂的基础上,以空白样品的加标回收率为指标,对S-MGCN用量、吸附时间、溶液pH、离子强度、洗脱溶液种类和体积等影响因素进行了优化,建立了基于S-MGCN的MSPE方法以提取淡水鱼中的MG及LMG,结合超高效液相色谱-串联质谱(UPLC-MS/MS),进行目标物的灵敏筛检。研究表明,S-MGCN对MG和LMG具有良好的吸附效率(94.2%以上),且净化样品基质效果好。该方法样品前处理简便,有机试剂的使用量少(5 mL),萃取时间短(2 min)。对两种目标物的检出限和定量限分别为0.075 μg/kg和0.25 μg/kg,灵敏度高于国标法(0.5 μg/kg);在0.25~20.0 μg/kg内线性关系良好(*r*>0.998),方法的回收率为88.8%~105.9%,日内和日间的相对标准偏差(RSD)均小于14%,准确度和精密度与国标法相当。最后,通过实际样品的检测验证了该方法的实际应用可行性。该文建立的基于S-MGCN的MSPE方法是一种高效环保的方法,为实际样品孔雀石绿和隐色孔雀石绿的灵敏筛检提供了新的方法学参考。

## Abstract (en)

Malachite green (MG) and its metabolite, leucomalachite green (LMG), exert toxic effects on the human body. The use of these dyes is illegal, but they are still detected in aquatic products. Freshwater fish are aquatic products with the high non-qualified rates. Therefore, the sensitive screening of MG and LMG in freshwater fish is of great importance to ensure the safety of aquatic products. Owing to the low contents of MG and LMG in fish and the complex matrix of actual samples, sample preparation is required before detection to purify impurities and enrich the target compounds. Graphite carbon nitride (GCN), a polymer material composed of C, N, and H, has good chemical and thermal stability, a large specific surface area, and a large number of active sites. It has a wide range of application prospects in adsorption and can be used in food safety testing when compounded with Fe_3_O_4_ to form magnetic graphite carbon nitride (MGCN). In this study, sulfonated magnetic graphite carbon nitride (S-MGCN) was prepared by further functionalizing MGCN with sulfonic acid. After characterization by scanning electron microscopy (SEM), Fourier transform infrared spectroscopy (FT-IR), and vibrating sample magnetometry (VSM), a magnetic solid-phase extraction (MSPE) method based on S-MGCN was established to extract MG and LMG from freshwater fish. The targets were screened using ultra performance liquid chromatography-tandem mass spectrometry (UPLC-MS/MS). Following sulfonic acid functionalization, S-MGCN showed increased electrostatic interactions based on the MGCN adsorption mechanism, which includes hydrogen bonds and *π-π* interactions; thus, its adsorption efficiency was significantly improved. The matrix effects were -42.21% and -33.77% before functionalization, -11.40% and -7.84% after functionalization, thus confirming that S-MGCN has significant matrix removal ability. Given that S-MGCN demonstrated excellent efficiency as an MSPE adsorbent, the adsorption conditions for S-MGCN were optimized. The optimal conditions were as follows: adsorbent dosage, 15 mg; adsorption time, 2 min; solution pH, 5; and ionic strength, not adjusted. Under these conditions, the adsorption efficiency of S-MGCN could reach 94.2%. Different organic solvents were used to elute adsorbed MG and LMG, and the desorption efficiency peaked when 1%(v/v) ammonia acetonitrile was used as the elution solvent. The elution volume was also optimized, and a maximum desorption efficiency of 93.2% was obtained when 1 mL of 1%(v/v) ammonia acetonitrile was added to S-MGCN. The limits of detection (LODs) and quantification (LOQs) of the two targets were determined at signal-to-noise ratios (*S/N*) of 3 and 10, respectively. The LODs and LOQs were 0.075 μg/kg and 0.25 μg/kg, respectively. The linear ranges of the two target compounds were 0.25-20.0 μg/kg with correlation coefficients (*r*) greater than 0.998. To assess accuracy and precision, we prepared spiked samples at three levels (low, medium, and high) with six parallel samples per level (*n*=6). The recoveries ranged from 88.8% to 105.9%. The intra- and inter-day relative standard deviations were 5.4%-13.7% (*n*=6) and 3.3%-11.1% (*n*=3), respectively. Compared with the national standard method, the proposed method features simpler sample pretreatment procedures, less use of organic reagents (5 mL), and a shorter extraction time (2 min); moreover, the method does not require complicated elution steps, and the eluent can be directly analyzed by UPLC-MS/MS. The test results of actual samples were consistent with those obtained via the national standard method, thus confirming the practical feasibility of the developed method. The proposed MSPE method based on S-MGCN is an efficient and environmentally friendly method that could provide a new methodological reference for the sensitive screening of MG and LMG in actual samples.

孔雀石绿(malachite green, MG)是一种严重威胁人体健康的致癌、致突变、致畸和呼吸毒性的三苯甲烷类化合物^[1,2]^,隐色孔雀石绿(leucomalachite green, LMG)是MG在生物体内的主要代谢产物,相对于MG,其在人体内半衰期更长,毒性更强^[3]^。虽然MG早在2003年就被列入了第一批“食品动物禁用的兽药及其他化合物清单”,禁止在所有食用动物中使用,且农业部公告第250号进一步将其列为禁止使用药物^[4]^,强调水产品中不得检出MG及其代谢产物LMG,但由于MG对鱼体和鱼卵的水霉病有特效,水产业养殖户常铤而走险,继续违规使用^[5]^。据2020年食用农产品市场监管部门抽检报告,淡水鱼为不合格率最高的水产品,共计597批次,其中MG检出70批次,检出率高达11.73%^[6]^。因此,淡水鱼中MG和LMG的灵敏筛检对水产品食用安全非常必要。

检测淡水鱼中的MG和LMG时,须进行样品前处理以净化样品基质和富集目标物。固相萃取(SPE)是最常使用的前处理方法^[7⇓-9]^,但在传统的SPE过程中,SPE吸附剂常为小粒径和非球形填料形式,因此会出现高背压或填料堵塞等问题^[10]^。磁性固相萃取(MSPE)是SPE的一种操作形式,吸附剂包括磁源(通常为Fe_3_O_4_)和非磁功能性材料两部分,磁源可与外部磁场相互作用,非磁功能材料可为目标物提供独特的吸附位点^[11]^。磁性吸附剂能够分散在大体积的样品中,增加吸附剂与目标物之间的接触机会,萃取效率高,且操作简便,仅需施加外部磁场即可实现目标物与样品溶液的分离,已广泛应用于食品安全分析、环境监测等领域^[12,13]^。Musevi等^[14]^制备了磁性氧化石墨烯可溶性蛋壳膜蛋白(Fe_3_O_4_/GO-SEP)作为MSPE吸附剂,通过静电引力、氢键作用及*π*-*π*相互作用等混合吸附机制,可在30 min内对水样中的MG完成萃取;Guo等^[15]^开发了使用油酸包覆的磁性纳米珠(OA-MNBs),在40 min内可完成对鱼肌肉组织中LMG的磁性固相萃取。以上研究说明基于纳米微球的MSPE能够简化LMG提取过程,但仍存在提取过程耗时较长,只能单一提取MG或LMG的问题。

氮化碳(graphite carbon nitride, GCN)是由C、N和H组成的一种高分子材料,具有良好的化学、热稳定性以及亲水亲油平衡性的三嗪结构^[16,17]^,且GCN本身含有丰富的-NH-和-NH_2_基团,利于该材料的功能化改性。目前已有基于磁性氮化碳(MGCN)或功能化MGCN的MSPE用于食品安全监测的研究报道。如,Li等^[18]^通过溶剂热法合成了MGCN,通过磁性固相萃取,达到了快速、简单地分析奶粉中雌激素的目的;Zhao等^[19]^采取水热合成法制备了一种三元纳米复合材料MGCN-MoS_2_,用作MSPE中的吸附剂,用于鸡肉和鸡蛋中痕量氟喹诺酮类药物的分离和浓缩。但尚未有MGCN或功能化MGCN应用于水产品中MG和/或LMG检测的研究报道。

本工作将GCN与Fe_3_O_4_复合,制得MGCN基底,通过氯磺酸与GCN上的-NH_2_基团反应引入磺酸基团,获得磺酸化磁性氮化碳(S-MGCN)作为吸附剂,通过优化基于S-MGCN的MSPE条件,结合超高效液相色谱-串联质谱(UPLC-MS/MS)建立了同时筛检淡水鱼样品中MG和LMG的方法,并通过对灵敏度、准确度、精密度等指标进行评价及对实际样品的检测验证了新方法的实际应用性能。

## 1 实验部分

### 1.1 仪器与试剂

UPLC超高效液相色谱仪与Xevo TQD三重四极杆质谱仪,配TQD质量检测器、Acquity自动采样器、ESI源(美国Waters公司); SX2-4-10A陶瓷纤维马弗炉(上海鳌珍仪器制造有限公司); BKT-4500型振动样品磁强计(北京新科高测科技有限公司); Zeiss Ultra Plus场发射扫描电子显微镜(scanning electron microscopy, SEM,德国Zeiss公司);傅里叶变换红外光谱仪(Fourier transform infrared spectroscopy, FT-IR,美国Nicolet公司); Heraeus Multifuge X1R型台式离心机(美国Thermo公司); Zetasizer Nano ZS90激光粒度仪(英国马尔文公司); DZF-6030真空干燥箱(上海飞越实验仪器有限公司);多头恒温磁力搅拌器(金坛区西城新瑞仪器厂); SHA-B水浴恒温振荡器(天津市赛得利斯实验分析仪器制造厂)。

所有试剂无特殊说明外均为AR级。三聚氰胺(纯度99%)、氯磺酸(ClSO_3_H,纯度为98%)、六水合三氯化铁(FeCl_3_·6H_2_O)、二氯甲烷(CH_2_Cl_2_)、乙二醇(EG)、乙酸乙酯、0.5 mol/L盐酸等均购自上海国药集团化学试剂有限公司;三水合乙酸钠(NaAc·3H_2_O)购自上海麦克林生化科技有限公司;乙腈和甲醇均为HPLC级,购自德国Merck公司。孔雀石绿(纯度98%)和隐性孔雀石绿(纯度98%)标准品均购自上海安谱实验科技股份有限公司。

准确称取两种目标物的标准品各10.0 mg,分别置于10 mL棕色容量瓶中,加入乙腈溶解并定容、摇匀,得到质量浓度为1000 mg/L的标准储备溶液。分别准确吸取MG和LMG的标准储备溶液各1.00 mL于100 mL棕色容量瓶中,用乙腈稀释得到含有两种目标物质量浓度均为10 mg/L的混合标准溶液。所有溶液均于-20 ℃避光保存。临用前准确吸取一定量的混合标准溶液,用超纯水稀释得到所需浓度的标准工作溶液。

### 1.2 S-MGCN的制备

将15 g三聚氰胺置于陶瓷纤维马弗炉中,以15 ℃/min的速率从25 ℃升温至550 ℃,维持4 h,待马弗炉冷却至室温后,得到GCN。

将0.4 g GCN超声均匀分散在60 mL的EG中,加入0.4 g FeCl_3_·6H_2_O和1.05 g NaAc·3H_2_O,于600 r/min转速下磁力搅拌30 min,将所得混合溶液转移至事先预热30 min的反应釜中,于200 ℃下反应12 h,收集反应产物,用2 mL超纯水和2 mL无水乙醇分别依次振荡洗涤3次,60 ℃真空干燥12 h,得到MGCN。

参照Zhang等^[20]^的方法修饰磺酸基团,将1 g MGCN放入带恒压滴液漏斗的三颈圆底烧瓶中,加入25 mL二氯甲烷,在恒温摇床上振荡均匀后,冰浴条件下,在20 min内经漏斗加入25 mL氯磺酸-二氯甲烷(1∶12.5, v/v)后,再次置于恒温摇床上,室温下以120 r/min振荡反应5 h。反应结束后,所得产物用2 mL乙酸乙酯和2 mL甲醇分别依次振荡洗涤3次,在60 ℃真空干燥8 h,得S-MGCN(见图1a)。

**图1:**
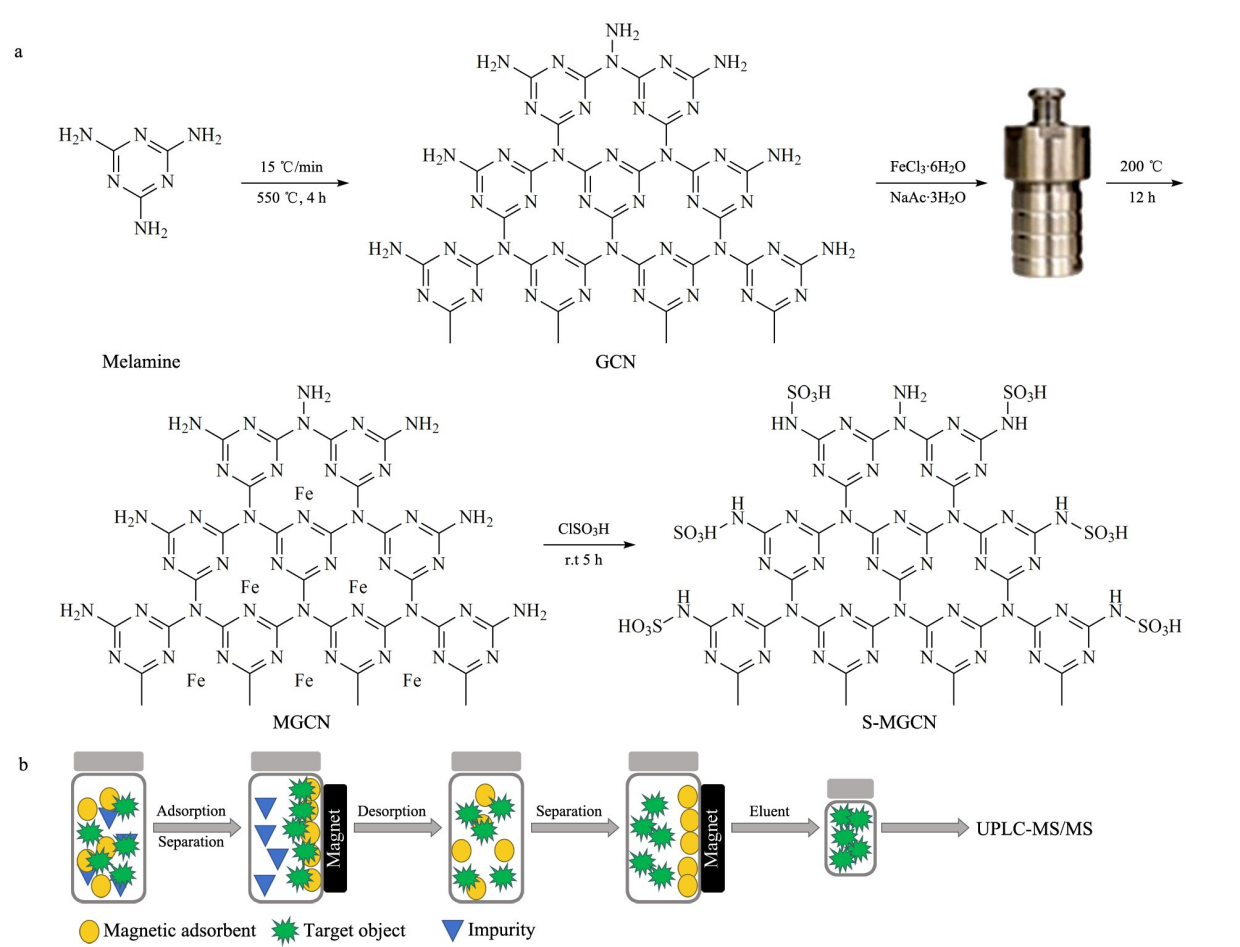
(a)S-MGCN材料的制备和(b)磁性固相萃取流程图

采用扫描电镜观察GCN和S-MGCN的形貌,测定GCN、MGCN以及S-MGCN的红外光谱,并对S-MGCN进行Zeta电位和磁强度分析。

### 1.3 样品的采集与处理

在苏州市姑苏区农贸市场采集淡水鱼样品。每条鱼分别取其背部、尾部及两面腹部的鱼肉各约15 g,共约60 g,混合后绞碎成鱼肉糜,置于100 mL样品瓶中,密封并对样品编号标记,于-20 ℃保存,一周内完成检测。

称取2 g(精确至0.01 g)室温下解冻的鱼肉糜样品于15 mL离心管中,向其中加入4 mL乙腈,涡旋萃取2 min后,以8000 r/min离心5 min,取200 μL上清液于另一15 mL离心管中,用超纯水稀释至4 mL,用0.5 mol/L的盐酸溶液调节pH至5,得到样品溶液。

在样品溶液中加入15 mg的S-MGCN,涡旋萃取2 min后,用磁铁将吸附剂与样品溶液分离,弃去上清液,用1 mL (0.5 mL+0.5 mL) 1%(v/v,下同)氨水乙腈将S-MGCN上吸附的目标物洗脱下来,取洗脱液直接进行UPLC-MS/MS分析(见图1b)。

### 1.4 UPLC-MS/MS分析

#### 1.4.1 色谱条件

色谱柱:ACQUITY UPLC BEH C18(100 mm×2.1 mm, 1.7 μm);柱温:40 ℃;流动相:A为0.1%甲酸水溶液(含2 mmol/L甲酸铵溶液),B为0.1%甲酸甲醇溶液;流速:0.3 mL/min。梯度洗脱程序:0~0.5 min, 70%A; 0.5~1.5 min, 70%A~35%A; 1.5~3.0 min, 35%A~15%A; 3.0~5.5 min, 15%A; 5.5~7.0 min, 15%A~70%A。进样量:3 μL。

#### 1.4.2 质谱检测参数

离子源:ESI源;扫描模式:正离子扫描模式;监测模式:多反应监测(MRM);离子源温度:150 ℃;脱溶剂温度:350 ℃。其他质谱参数见表1。

**表1:** 两种目标物的质谱参数

Compound	*t*_R_/ min	Precursor ion (*m/z*)	Product ion (*m/z*)	CV/ V	CE/ eV
Malachite green	2.53	329	313^*^	50	30
(MG)			208		35
Leucomalachite	3.90	331	316^*^	40	20
green (LMG)			239		30

* Quantitative ion. CV: cone voltage; CE: collision energy.

### 1.5 S-MGCN作为MSPE吸附剂的评价

#### 1.5.1 对目标物的吸附效率

以未检出目标物的淡水鱼样品作为空白样品,按1.3节进行处理,得到空白样品溶液,在其中加入一定体积的标准工作溶液,得到MG和LMG质量浓度均为2.0 μg/L的基质匹配工作溶液。分别加入15 mg的MGCN或S-MGCN,涡旋萃取2 min,用磁铁将吸附剂与样品溶液分离,弃去上清液,分别用1 mL 1%氨水乙腈将MGCN或S-MGCN上吸附的目标物洗脱下来,取洗脱液直接进行UPLC-MS/MS分析。以回收率为指标,评价MGCN和S-MGCN对目标物的吸附效率。回收率(Recovery)按式(1)计算:


(1) $$\text{ Recovery }=\frac{CV}{C_{0}V_{0}}\times 100\%$$

式中*C*_0_(2.0 μg/L)为各目标物在基质匹配工作溶液中的质量浓度,*C*(μg/L)为洗脱溶剂中各目标物的质量浓度,*V*(1 mL)为洗脱溶剂的体积,*V*_0_(4 mL)为基质匹配工作溶液的体积。

#### 1.5.2 对样品基质的净化效果

分别将2.0 μg/L的基质匹配工作溶液与同浓度的标准工作溶液进行UPLC-MS/MS分析,分别测得其中目标物的峰面积,按式(2)计算各目标物在MSPE处理前的基质效应(M ${E_{before}}_{MSPE}$ )。


(2) $${ME}_{\text{before MSPE }}=\frac{A_{1}-A_{0}}{A_{0}}\times 100\%$$

式中*A*_1_为2.0 μg/L基质匹配工作溶液中各目标物的峰面积,*A*_0_为同浓度标准工作溶液中各目标物的峰面积。

将空白样品按1.3节进行处理至得到洗脱液,在洗脱液中加入一定体积的标准工作溶液,得到MG和LMG质量浓度均为2.0 μg/L的进样溶液。分别将进样溶液与同浓度的标准工作溶液进行UPLC-MS/MS分析,分别测得其中目标物的峰面积,按式(3)计算各目标物经MSPE处理后的基质效应( ${{\mathrm{ME}}_{after}}_{MSPE}$ )。


(3) $${ME}_{\text{after MSPE }}=\frac{A_{2}-A_{0}}{A_{0}}\times 100\%$$

式中*A*_2_为2.0 μg/L进样溶液中各目标物的峰面积。

通过比较各目标物进行MSPE前、后的基质效应,评价基于S-MGCN的MSPE对样品基质的净化效果。

### 1.6 方法学评价试验

为更好地与实际应用相匹配,本工作采用模拟样品进行方法的各效能指标的评价。

#### 1.6.1 线性范围、检出限和定量限

在2 g(精确至0.01 g)空白样品中加入10 μL不同浓度的标准工作溶液,涡旋混匀20 s,分别得到MG和LMG含量均为0.25、0.5、1.0、2.5、5.0、10.0、20.0 μg/kg的模拟样品,各模拟样品按1.3节进行前处理之后,按1.4节进行UPLC-MS/MS分析,测得各目标物的峰面积。对各目标物的峰面积和模拟样品中相应的含量进行线性拟合,得到MG和LMG的工作曲线。

#### 1.6.2 准确度和精密度

在2 g(精确至0.01 g)空白样品中加入10 μL不同浓度的标准工作溶液,涡旋混匀20 s,分别得到低、中、高(0.5、2.5和10.0 μg/kg)3个加标水平的模拟样品,每个水平分别制备6个平行样(*n*=6)。各模拟样品按1.3节进行前处理之后,按1.4节进行UPLC-MS/MS分析,测得各目标物的峰面积,分别带入工作曲线计算得到MG和LMG的含量,以测得含量与相应的低、中、高3个加标水平的比值计算得到的相对回收率评价准确度;以1 d内和连续3 d低、中、高3个加标水平的模拟样品中各目标物测得含量的相对标准偏差(RSD)分别计算得到日内和日间精密度。当RSD小于15%时,表明结果为可接受范围^[21]^。

## 2 结果与讨论

### 2.1 S-MGCN的表征

GCN、MGCN和S-MGCN的场发射扫描电镜表征如图2所示,可见GCN为典型的片状结构,MGCN为Fe_3_O_4_球形颗粒覆盖在GCN表面,S-MGCN与MGCN的外观在电镜下无明显差别。GCN、Fe_3_O_4_、MGCN、S-MGCN的傅里叶红外光谱分析结果如图3所示, S-MGCN在540.4、811.4、992.6、1205.7、1623.2、3173.7 cm^-1^处出现了强吸收峰,分别对应Fe-O键、三嗪结构、S-O键、O=S=O键、C-N键、N-H键,说明S-MGCN材料制备成功。如图4所示,S-MGCN的Zeta电位测定结果表明S-MGCN表面带有负电荷。S-MGCN的磁化饱和度值为22.02624 emu/g, 15 s内可被磁铁完全吸附,满足MSPE需求。

**图2:**
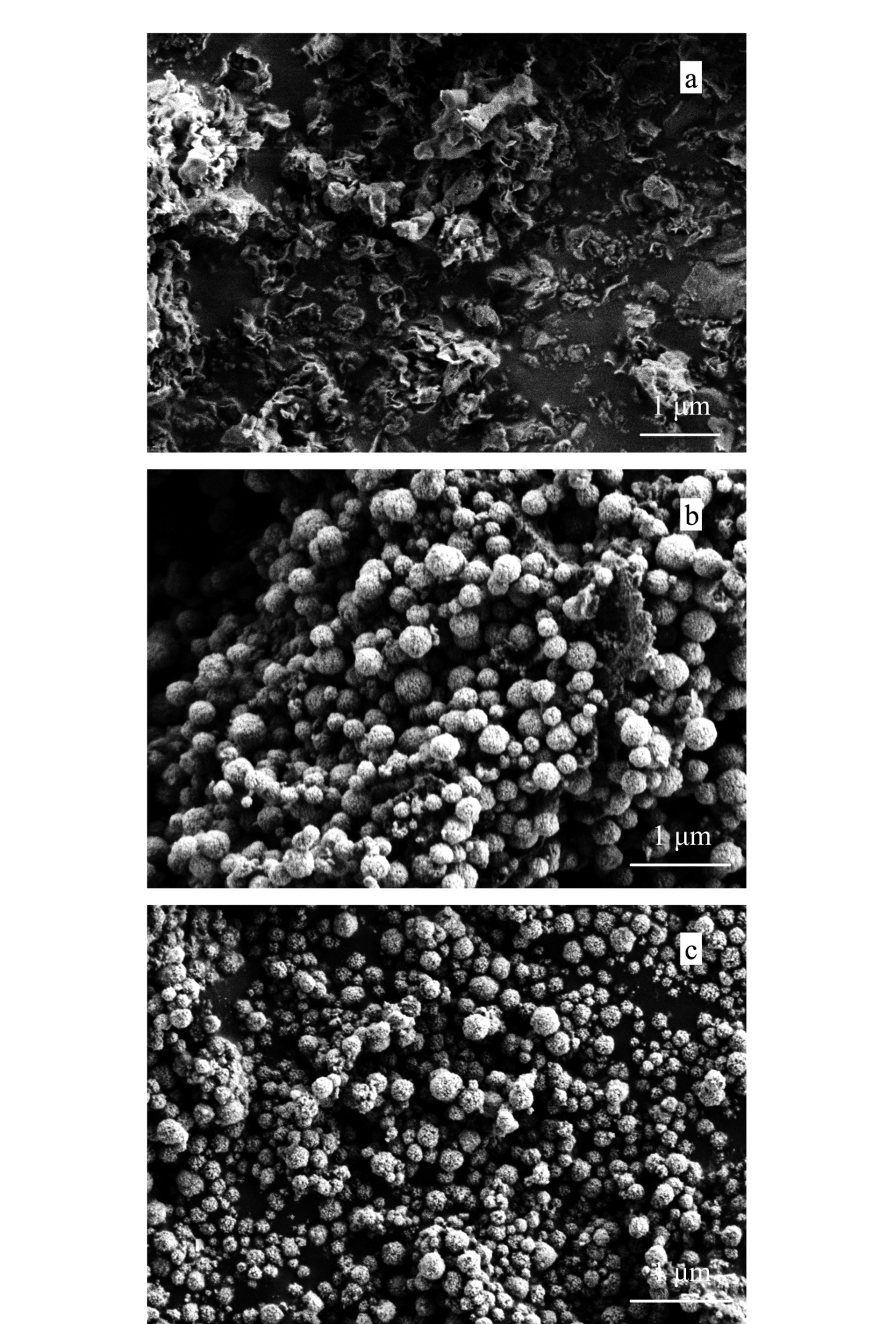
(a)GCN、(b)MGCN和(c)S-MGCN的扫描电镜图

**图3:**
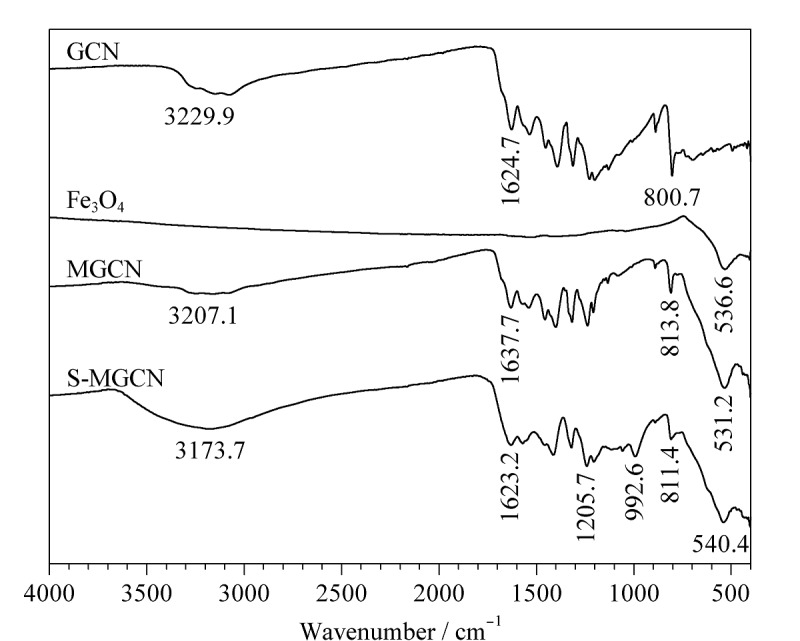
GCN、Fe_3_O_4_、MGCN、S-MGCN的傅里叶红外光谱图

**图4:**
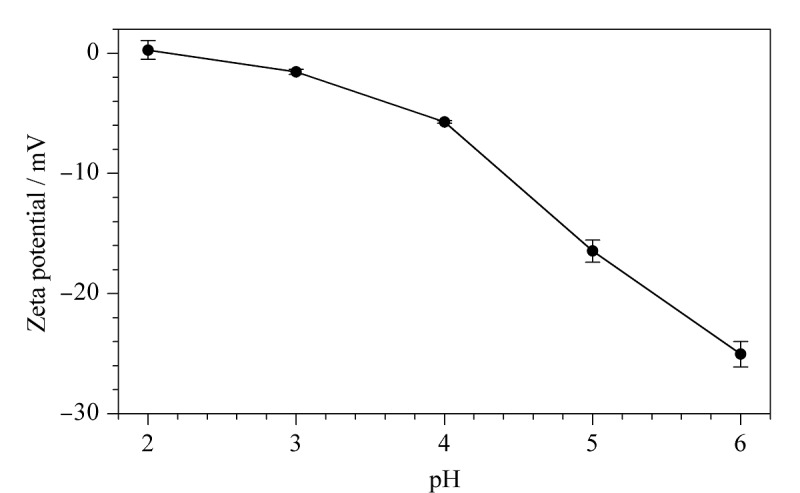
S-MGCN的Zeta电位图

### 2.2 S-MGCN作为MSPE吸附剂的评价

对MGCN和S-MGCN两种吸附剂的吸附效果进行比较,结果如图5所示,S-MGCN对目标物的吸附效果对比MGCN有明显提升,表明修饰的磺酸基团在吸附过程中发挥了良好的吸附作用。由S-MGCN的Zeta电位图可以看出,溶液pH在2~6时,S-MGCN表面带有负电荷。MG和LMG的p*K*_a_分别为6.90和5.62,当溶液pH小于目标物的p*K*_a_时,两种目标物可质子化而带正电,可以与表面带有负电荷的S-MGCN产生静电引力。MGCN的三嗪结构以及富含的氨基官能团可分别与目标物形成*π-π*相互作用和氢键,在此基础上,S-MGCN修饰的磺酸基团又增加了与目标物之间的静电引力,进一步丰富了吸附作用机制,因此S-MGCN对目标物的吸附效能优于MGCN。

**图5:**
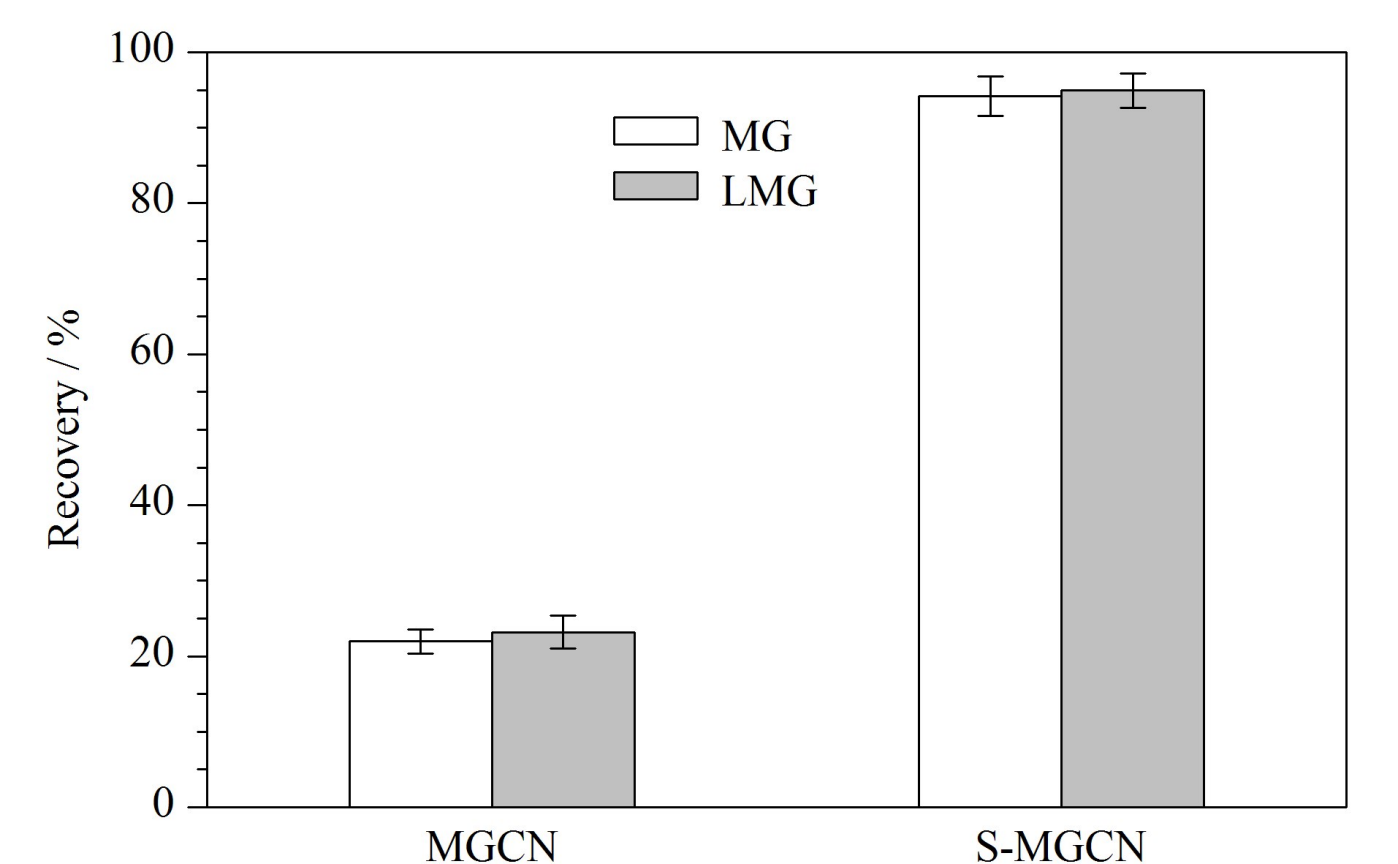
MGCN和S-MGCN对2种目标物的吸附效率(*n*=3)

基质效应结果表明,MSPE前基质效应为-42.21%和-33.77%,而经过基于S-MGCN的MSPE后,两种目标物的基质效应降低至-11.40%和-7.84%。另外,通过比较MSPE前后加标淡水鱼样品中两种目标物的提取离子流色谱图(见图6)可见,目标物经MSPE处理后响应值明显提高,杂质峰明显降低。表明基于S-MGCN的MSPE能有效净化样品基质。

**图6:**
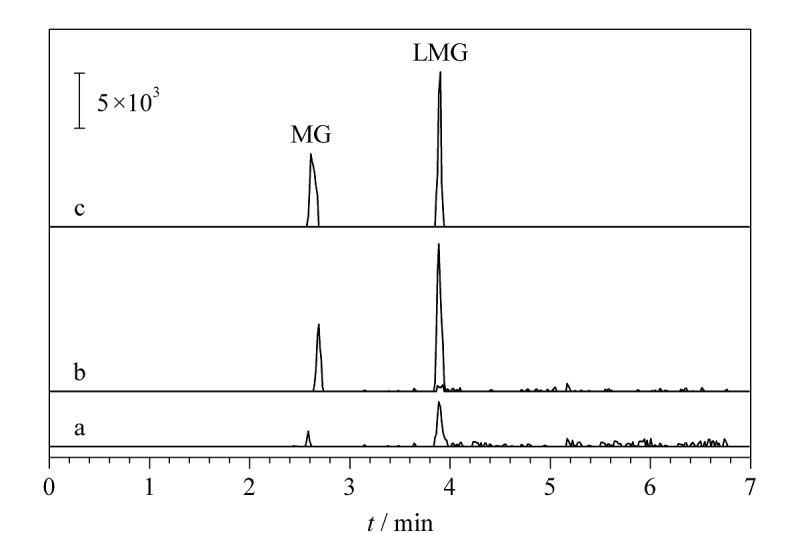
2种目标物的提取离子流色谱图

### 2.3 MSPE影响因素的考察及条件优化

将空白样品按1.3节进行处理,得到空白样品溶液,在其中加入一定体积的标准工作溶液,得到MG和LMG质量浓度为2.0 μg/L的基质匹配工作溶液,以回收率为指标,对MSPE的影响因素进行优化,包括S-MGCN的用量、溶液pH值、吸附时间、离子强度、洗脱溶剂类型和洗脱溶剂体积等,回收率按式(1)计算。MSPE影响因素的考察及条件优化结果如图7所示。

**图7:**
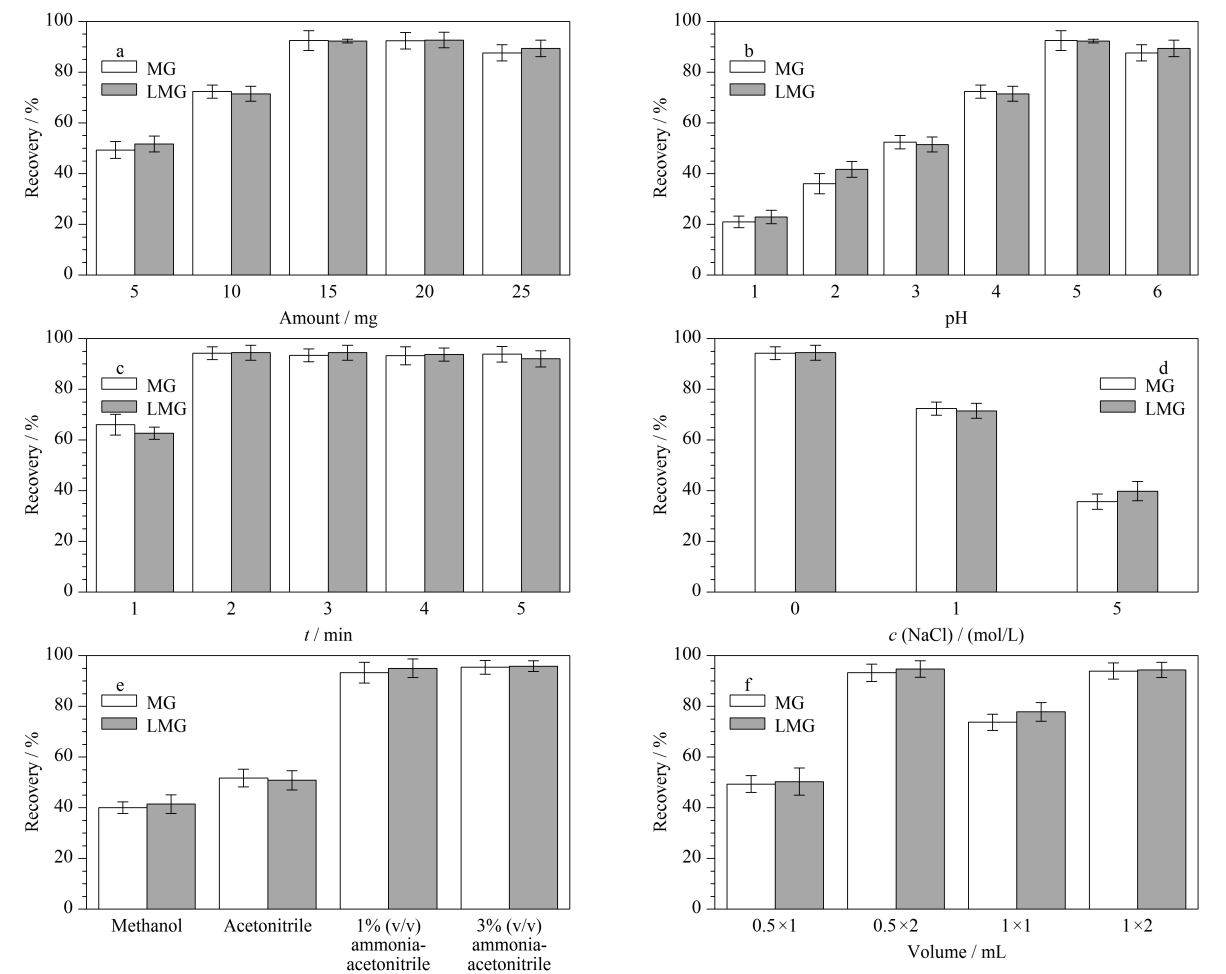
(a)吸附剂的使用量,(b)溶液pH, (c)吸附时间,(d)样品离子强度,(e)洗脱溶剂,(f)洗脱溶剂体积 (单次洗脱体积×洗脱次数)对2种目标物萃取效率的影响(*n*=3)

由图7a可见,采用5~15 mg S-MGCN时,回收率呈上升趋势;继续增加S-MGCN用量至20 mg,吸附效率无明显提升;而当用量达到25 mg时,回收率略有降低,可能是当吸附剂的用量过多时会产生团聚作用,导致吸附位点减少。说明15 mg S-MGCN足够用于吸附目标物。

考察了样品溶液pH值(pH 1~6)对MG和LMG萃取效率的影响。如图7b所示,当溶液pH值为5时,S-MGCN对MG和LMG的回收率最高,原因可能是此时S-MGCN与MG、LMG之间的静电作用力最大。如2.2节所述,两种目标物可质子化而带正电,从而与表面带有负电荷的S-MGCN产生静电引力。由图4可知,pH值为2~5时,此时目标物可质子化而带正电,而S-MGCN表面带有的负电荷逐渐增多,与目标物之间的静电引力增加,有利于吸附;当pH>5时,S-MGCN表面带有的负电荷虽继续增多,但随着溶液pH增大,目标物质子化效率低,S-MGCN无法对溶液中的目标物进行有效吸附。因此,调节样品溶液pH为5后进行MSPE处理。

涡旋吸附时间对吸附效率的影响如图7c所示,可见MG和LMG在S-MGCN上可快速达到吸附平衡。当吸附时间小于2 min时,S-MGCN对MG和LMG的吸附并未到达吸附平衡,没有实现完全吸附,因此吸附效率并未达到最高;当吸附时间为2 min时,回收率即可达94.2%,继续增加吸附时间未见吸附效率显著提升;而当吸附时间达到5 min,由于吸附时间过长,MG与LMG可能存在脱附的情况,导致吸附效率有轻微下降。因此,吸附时间选择为2 min。

本工作在样品溶液中添加一定浓度的NaCl以考察离子强度对S-MGCN吸附目标物的影响。如图7d,当NaCl浓度从0增加到5 mol/L时,两种目标物的回收率显著降低。这可能是因为添加的NaCl破坏了MG、LMG与S-MGCN之间的静电引力,导致吸附效率下降。因此,在MSPE过程中无需调节离子强度。

本工作还考察了甲醇、乙腈、1%氨水乙腈和3%氨水乙腈对S-MGCN上所吸附目标物的洗脱效能。如图7e所示,采用1 mL的1%氨水乙腈或3%氨水乙腈时,目标物回收率即可达93.2%以上,且二者回收率无明显差异,故本工作选择1%氨水乙腈作为洗脱溶剂。随后即优化了洗脱剂的体积和洗脱次数,如图7f所示,使用相同体积洗脱液时,进行两次洗脱较单次洗脱回收率更高,而使用1 mL或2 mL洗脱溶剂分两次进行洗脱时(每次0.5 mL或1 mL), MG和LMG的回收率均可达93.2%以上,且二者回收率无明显差异。因此,本工作选择采用1 mL的1%氨水乙腈对S-MGCN吸附的目标物分两次进行洗脱,每次用0.5 mL,合并洗脱液直接进行UPLC-MS/MS分析。

### 2.4 方法学验证结果

如表2所示,本方法在MG和LMG含量为0.25~20.0 μg/kg内表现出良好的线性关系,相关系数(*r*)均大于0.998。以信噪比(*S/N*)=3和*S/N*=10确定样品中MG和LMG的检出限(LOD)和定量限(LOQ),分别为0.075 μg/kg和0.25 μg/kg,与国标中MG和LMG的LOD(0.5 μg/kg)^[22]^相比,本方法的LOD更低,具有更好的灵敏度。

**表2:** 2种目标物的线性范围、线性方程、相关系数、 检出限和定量限

Compound	Linear range/ (μg/kg)	Linear equation	*r*	LOD/ (μg/kg)	LOQ/ (μg/kg)
MG	0.25-20.0	*y*=224.37*x*+3.6060	0.9983	0.075	0.25
LMG	0.25-20.0	*y*=123.40*x*-0.4229	0.9997	0.075	0.25

*y*: peak area; *x*: spiked level, μg/kg.

为保证方法的准确性和重复性,需对方法的准确度和精密度进行考察,根据方法建立和应用时的实际情况,需进行不同时间的测量,即同一工作日内的不同时间和不同的工作日。实验过程中的偶然误差,如仪器正常波动、实验室环境(如温度、湿度)的变化及工作人员在实验操作过程中的微小差别等,常导致测得数据并不完全相同,直接体现在方法的精密度上。而只有当精密度符合要求时,对方法的准确度评价才有意义。如表3所示,低、中、高3个加标水平的模拟样品中,两种目标物的相对回收率为88.8%~105.9%;日内及日间精密度均小于14%,表明本方法具有良好的准确度和精密度。

**表3:** 淡水鱼中2种目标物的回收率和精密度

Compound	Spiked level/ (μg/kg)	Recovery/ %	RSDs/%	
			Intra-day (*n*=6)	Inter-day (*n*=3)
MG	0.5	88.8	13.7	3.3
	2.5	105.6	6.3	11.1
	10.0	88.8	7.2	3.3
LMG	0.5	97.4	10.9	3.3
	2.5	96.5	5.4	10.4
	10.0	105.9	9.1	6.6

### 2.5 与其他方法的比较

将本方法与国标方法(GB/T 19857-2005)进行比较,本方法只需15 mg S-MGCN即可完成对目标物的吸附,无需淋洗即可洗脱,且收集的洗脱液无需旋转蒸发、复溶及过滤,即可直接进行UPLC-MS/MS分析。本方法不仅简化了样品前处理过程,还减少了有机试剂的用量,仅为国标方法中有机试剂用量(30 mL)的1/6。

将本方法与文献[15,23⇓⇓⇓⇓⇓⇓⇓-31]中的方法进行比较(文献检索策略:关键词为水产品、孔雀石绿、隐色孔雀石绿、固相萃取;检索年份为2006-2022;检测数据库为知网和Web of Science)。结果如表4所示,本方法的LOD及回收率与列表文献方法相当或更优,且样品前处理时所需的有机试剂和吸附剂用量更少(5 mL和15 mg)、SPE提取时间更短(2 min),说明本方法不仅可用于淡水鱼中MG和LMG的同时灵敏筛查,而且操作简便、快速。

**表4:** 本方法与其他方法的比较

Samples (amount)	Detection method	Adsorbents (amount)	SPE time/ min	Reagent volume/ mL	LODs/ (μg/kg)	LOQs/ (μg/kg)	Recoveries/ %	Ref.
Freshwater fish (0.5 g)	LC-MS/MS	OA-MNBs (6 mg)	10	3.3	0.100	0.350	71.2-112.6	[15]
Freshwater fish (5.0 g)	HPLC-UV	MMIPs (50 mg)	120	12.0	0.500-0.600	1.70-2.00	88.9-102.0	[23]
Freshwater fish, prawn (5.0 g)	LC-MS/MS	MCX (60 mg)	-	110.0	0.014-0.018	0.0480-0.0600	75.0-95.0	[24]
Freshwater fish (5.0 g)	LC-MS/MS	QuEChERs (200 mg)	5	10.0	0.600	2.00	63.0-112.0	[25]
Freshwater fish (5.0 g)	UPLC-MS/MS	EMR-Lipid (1000 mg)	5	10.0	0.200	0.500	77.1-106.6	[26]
Freshwater fish, prawn (4.0 g)	HPLC	MIP (50 mg)	-	6.0	0.050-0.100	0.170-0.350	30.6-73.9	[27]
Freshwater fish (1.0 g)	UPLC-MS/MS	G (10 mg)	2	17.0	0.075	0.250	82.0-103.4	[28]
Freshwater fish, prawn (2.5 g)	UPLC-MS/MS	QuEChERs (650 mg)	8	10.0	0.101-0.133	0.337-0.443	83.3-110.0	[29]
Freshwater fish, prawn (5.0 g)	UPLC-MS/MS	QuEChERs (140 mg)	-	35.0	0.100-0.200	0.330-0.670	70.0-120.0	[30]
Freshwater fish (5.0 g)	UPLC-MS/MS	QuEChERs (1100 mg)	11	10.0	0.500	1.60	98.1-127.0	[31]
Freshwater fish (2.0 g)	UPLC-MS/MS	S-MGCN (15 mg)	2	5.0	0.075	0.250	88.8-105.9	this work

OA-MNBs: oleic acid-coated magnetic nanobeads; MMIPs: magnetic molecularly imprinted polymer; MCX: strong cation exchange column; EMR-Lipid: enhanced matrix removal lipid; MIP: molecularly imprinted polymer; G: graphene.

### 2.6 实际样品检测

从苏州市姑苏区农贸市场采集10份淡水鱼样品,按照1.3节和1.4节进行前处理和分析,每份做3个平行样。结果表明:在1个样品中检测到LMG,残留量为(0.55±0.02) μg/kg,与采用国标方法(GB/T 19857-2005)得到的检出情况一致(测得水平为(0.54±0.04) μg/kg),无统计学差异(*p*>0.05),表明本方法实际应用可行。

## 3 结论

本研究成功制备了复合吸附材料S-MGCN,并基于S-MGCN材料的MSPE结合UPLC-MS/MS建立了同时测定淡水鱼中MG和LMG的新方法。S-MGCN的三嗪结构、富含的氨基官能团、修饰的磺酸基团可分别与目标物形成*π-π*相互作用、氢键及静电引力等多种相互作用机制,可高效吸附目标物,对复杂的样品基质具有良好的净化能力。方法的灵敏度、准确度和精密度与国家标准相当或更优,且简化了样品前处理过程。同时,基于其丰富的吸附机制,S-MGCN可能对含有芳杂环、羟基、氨基等官能团或其他碱性分析物也有较好的吸附效能,具有良好的应用潜力。
